# Another amazing year for our federation

**DOI:** 10.4103/0972-0707.71639

**Published:** 2010

**Authors:** Anil Chandra

**Affiliations:** President - FODI and Professor, Department of Conservative Dentistry and Endodontics, Dental faculty, CSM Medical University (K. G. M. C.), Lucknow. E-mail: ahanachandra@yahoo.com

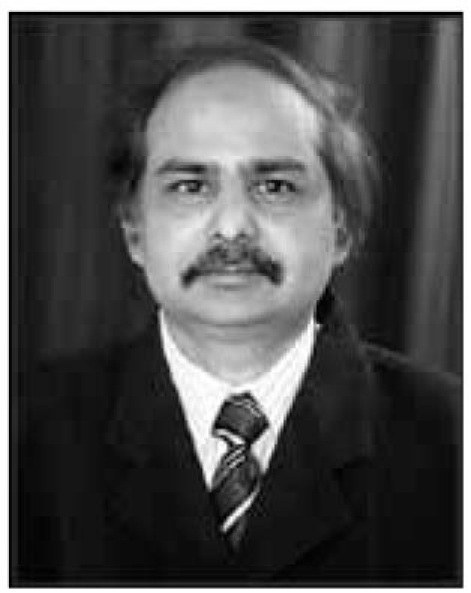



It seems not so long ago that we were gathered in Cochin, attending the exceptional scientific sessions, meeting old friends (adding new ones), and celebrating the success of our incredible Federation of Operative Dentistry of India. Our past presidents highly acclaimed performance will always be remembered.

All the federation’s existing initiatives and developments are a product of the hard work and dedication of our ever smiling secretary Dr. Lakshmi Narayanan L, under the able guidance of the DCI president, our own, Padma Bhushan Dr. Anil Kohli. Without a doubt the success we experienced in the past year would not have been possible unless we had his guidance and help. The highlights of this year’s achievements include two major factors, besides many more.

- The release of Grossman’s Endodontic Practice – Twelfth International Edition, which has been edited by two Indian authors, which makes this a remarkable achievement for Indian Endodontics, This classic textbook is co-edited by Dr. B. Suresh Chandra, a senior member of our Federation and Dr. V. Gopikrishna, the youth icon of Endodontics, who holds promise for a bright future for Endodontics in our country. We all join together in wishing Dr. B. Suresh Chandra a speedy recovery from his recent illness, with a hope that he will continue to spread the light of knowledge in the years to come.
- The other commendable achievement has been the indexing of our National Journal of Conservative Dentistry with Pubmed, due to the untiring efforts of our dynamic Editor-in-chief — Dr. V. Gopikrishna and his editorial team. I take this opportunity to congratulate him on behalf of our federation. This ensures that the scientific output of our federation reaches a global audience.

For my young friends in the Federation and profession, I can only say, be confident, have a positive attitude, dream big, and visualize success. In today’s technologically enhanced and highly competitive world, it is imperative not only to stay current, but also to stay ahead. If you are doing today what you did yesterday, then you are a day behind, therefore, we all have to be involved in the process of continuing education or perhaps we can call it ‘CANI’, that is, *Continuing And Never Ending Improvement*.

My best wishes to you all and I wish that everything you do far exceeds your expectations, always.

